# A primer on current status and future opportunities of clinical optoacoustic imaging

**DOI:** 10.1038/s44303-024-00065-9

**Published:** 2025-01-27

**Authors:** Ferdinand Knieling, Serene Lee, Vasilis Ntziachristos

**Affiliations:** 1 Department of Pediatrics and Adolescent Medicine, University Hospital Erlangen, Erlangen, Germany; 2 Chair of Biological Imaging, Central Institute for Translational Cancer Research (TranslaTUM), School of Medicine and Health & School of Computation, Information and Technology, Technical University of Munich, Munich, Germany; 3 Institute of Biological and Medical Imaging, Bioengineering Center, Helmholtz Zentrum München, Neuherberg, Germany

**Keywords:** Medical imaging, Imaging

## Abstract

Despite its introduction in the 1970’s, it is only recent technology advances that have propelled growth in clinical optoacoustic (photoacoustic) imaging over the past decade. We analytically present the broad landscape of clinical optoacoustic applications in the context of these key technology advances, the unique contrast achieved, and the tissue biomarkers resolved. We then discuss current challenges and future opportunities to address the unmet clinical needs.

## Introduction

Optoacoustic (OptA) imaging, also termed photoacoustic imaging, represents a quantum leap in optical imaging. Optical methods have been used since the origin of medicine to visually inspect a patient and were later based on endoscopy and spectroscopy approaches. However, optical measurements have been limited by photon scattering, which has notoriously challenged accurate measurements and high-fidelity imaging deeper than 1 mm. Using light of transient energy, typically light pulses, to excite ultrasound waves within tissue, OptA imaging transforms the optical method to a high-resolution modality by resolving optical contrast based on ultrasonic diffraction. Transient light absorbed by tissue generates a minute temperature change in volumes that contain chromophores, which leads to a local volumetric expansion and contraction, known as the thermoelastic effect. The ultrasound waves generated are detected non-invasively on the tissue surface and are used in a mathematical inversion to reconstruct images of the optical contrast that generated them. A unique feature of OptA imaging is that depending on the wavelength(s) of light employed, different tissue chromophores can be resolved based on their spectral absorption characteristics at high resolution. This ability leads to imaging of oxygenated and deoxygenated hemoglobin, vasculature, blood oxygen saturation, oxygen demand, melanin, lipids, and other moieties without the use of contrast agents.

Optoacoustic imaging technology first appeared in the late 1970’s^1,2^ but it took another 2 decades to demonstrate the first in vivo human images^3^, primarily focusing on resolving vasculature in breast cancer based on hemoglobin contrast. Possibly due to the size and complexity of the illumination and detection methods required and the need to develop elaborate mathematical approaches for high-fidelity image formation with clinically relevant contrast, optical imaging using diffuse photons initially saw a much faster growth in biomedical applications in the 1990’s and 2000’s than OptA methods, despite the significantly compromised imaging performance due to photon scattering. However, technology maturation in the mid-2010’s propelled a consistent growth in the clinical application of the OptA modality. Yet there is no report that analyzes the type of studies, indications, performance, and technology in clinical optoacoustics or a perspective that links the unique capabilities offered by the modality to unmet clinical needs.

In this review, we take a critical look at the progress that has been achieved over the past decade in the field of optoacoustics and identify 370 studies on OptA imaging in humans. Many of these studies examine technical feasibility; however, since 2015, attention has increasingly shifted to exploiting the unique OptA contrast to introduce new potential for clinical application, not possible by current radiology methods. Conversely, critical challenges for clinical dissemination remain. To explore the field and present the unique ability and progress achieved over the past decade, and also the remaining barriers, we summarize all in-human studies identified and report them as a function of time to showcase the increasing interest and growth. We classified the studies based on the number of participants, to separate pilot studies from studies with statistical relevance. We further analyzed the most prevalent indications and clinical findings, the mode of operation, and key technology advances that have generated this recent interest for clinical use. We discuss the unique features of the modality as the basis for introduction in clinical routine and outline the challenges and outlook. We aim for this review paper to serve as the basis for understanding clinical optoacoustics and motivating clinical translation.

## Optoacoustic studies in humans

We interrogated the PubMed and Web of Science databases with the broad search terms in Table 1. The search did not use a restriction on publication year. For all papers, we identified the particular indication and disease, medical field (dermatology, oncology, etc.), contrast and target, key finding/s, number of participants imaged (patients, controls, or healthy volunteers), frequency of ultrasound detection (MHz), excitation wavelength/s (nm), and the OptA technique employed. Studies on human tissues ex vivo were not included in the analysis.

**Table 1:** Search terms used to interrogate the PubMed and Web of Science databases for articles on optoacoustic imaging in humans

Search operator	Search term
OR	Optoacoustic imaging
OR	Photoacoustic imaging
OR	Optoacoustic images
OR	Photoacoustic images
AND	Participant OR volunteer OR patient

Each paper was cross-checked with the entire list of publications identified to avoid duplicate entries. Cross-checking was particularly necessary, especially since there is no standardized terminology for the OptA modality, and many papers may use both terms optoacoustics and photoacoustics, whereas others only refer to the technology by one of the terms. Nevertheless, in analogy to using the term *optical* in optical imaging, optical microscopy, optical spectroscopy, optical coherence tomography, and so on, we use herein the term “optoacoustic” throughout the review, not “photoacoustic”. Optics, from the Greek word *οπτική* refers to the field that uses light for visualization. This notation is better suited to describe an imaging modality, and this is why the term optical is employed for virtually all imaging modalities using light. Photonics, from the Greek word *φως,* meaning light, is typically considered a sub-category of optics that uses the particle aspect of light or studies the fundamental principles of photons. We also note that although we use the abbreviation “OptA” to denote the term optoacoustic throughout this review, the abbreviations “OA” for optoacoustics and “PA” for photoacoustics have also been commonly employed in the literature. Nevertheless, we prefer “OptA” as it is more specific than OA and PA, and its pronunciation is closer to the word optoacoustics.

**Fig. 1:**
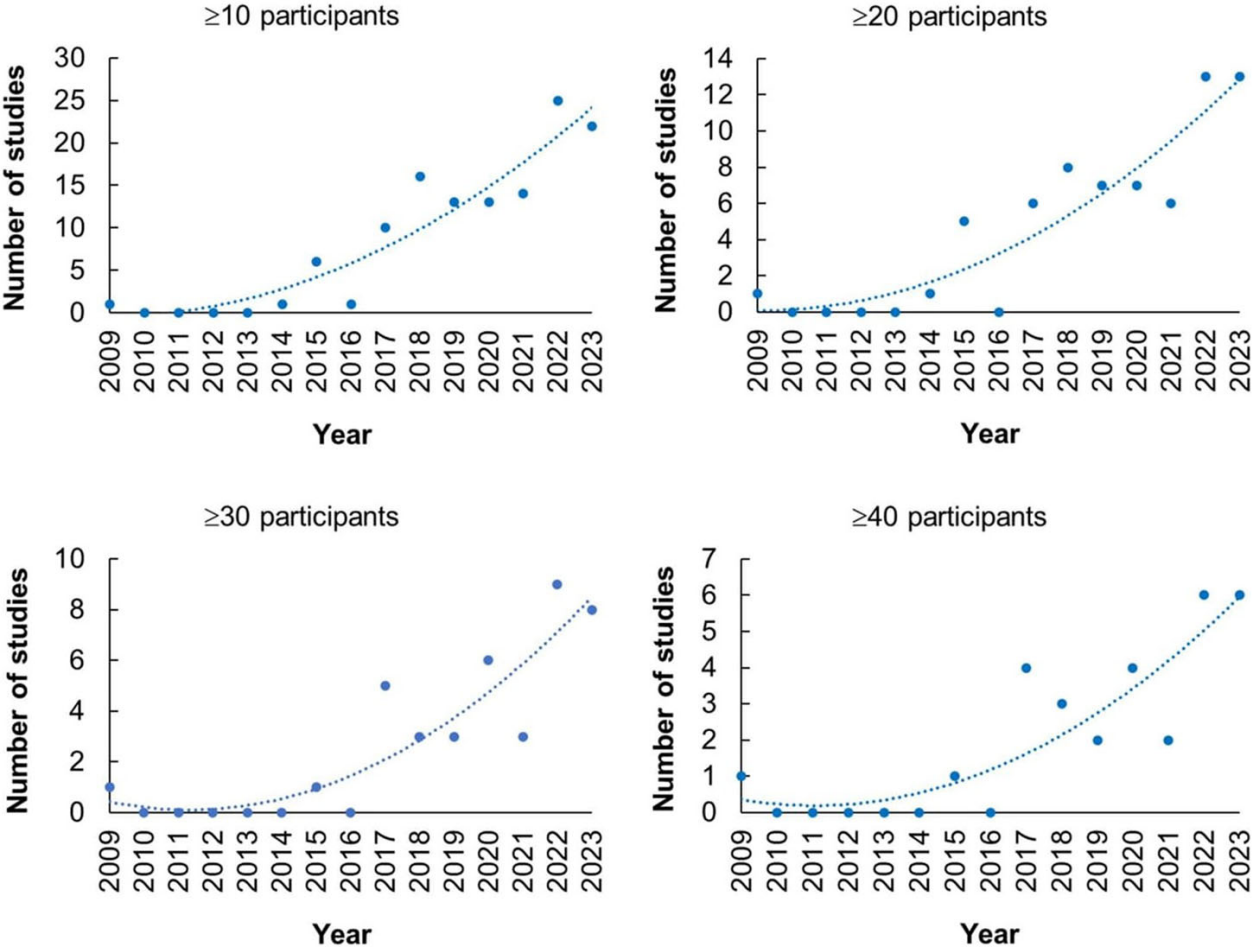
Number of in vivo optoacoustic studies with various numbers of participants.

Our search identified 370 publications on OptA imaging in humans. Many of these studies go well beyond technical demonstrations or feasibility studies and demonstrate a constant growth of in-human optoacoustics (Fig. 1 and Supplementary Fig. 1). When examining studies with more than 10 participants, the volume of publications appears to start growing in the years 2014–2015 and beyond, a trend that remains when examining studies with more than 20, 30, or 40 participants (Fig. 1 and Supplementary Fig. 1b) or studies with 10–19, 20–29 or 30–39 participants (Supplementary Fig. 1c). The graphs show that a consistent growth of OptA studies in humans and with increasing numbers of participants are indeed no more than 10 years old.

**Fig. 2:**
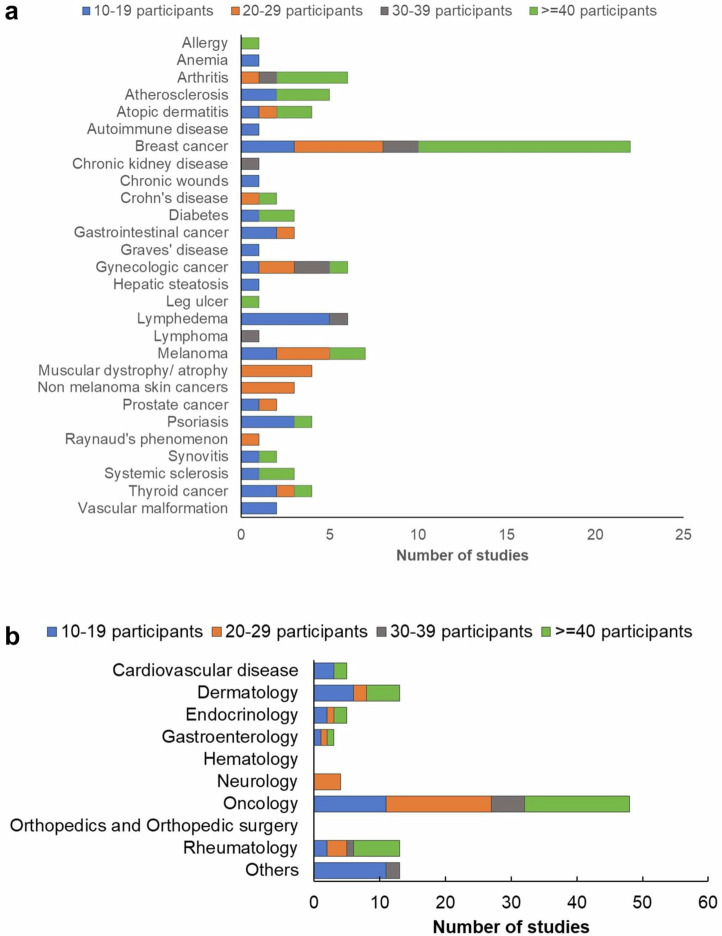
Optoacoustic imaging in different indications and medical fields. **a** Indications examined in humans in vivo with optoacoustic imaging, and **b** the number of studies split across different medical fields.

Summarizing the indications studied, we find that the leading indication is breast cancer (Fig. 2a). Other notable indications include arthritis, gynecological cancers, atherosclerosis, and melanoma. Atopic dermatitis, psoriasis, muscular dystrophy, and thyroid cancer also appear in 4 studies, each with more than 10 participants. Therefore, optoacoustics has been employed with priority in oncology applications, followed by rheumatology and dermatology (Fig. 2b). About 220 of all studies identified are only on healthy volunteers, whereas 150 studies recruited patients or participants with certain conditions (Supplementary Fig. 2). Thirty-four studies were performed with ≥40 participants and 14 had ≥100 participants.

## Optoacoustic macroscopy and mesoscopy

**Fig. 3:**
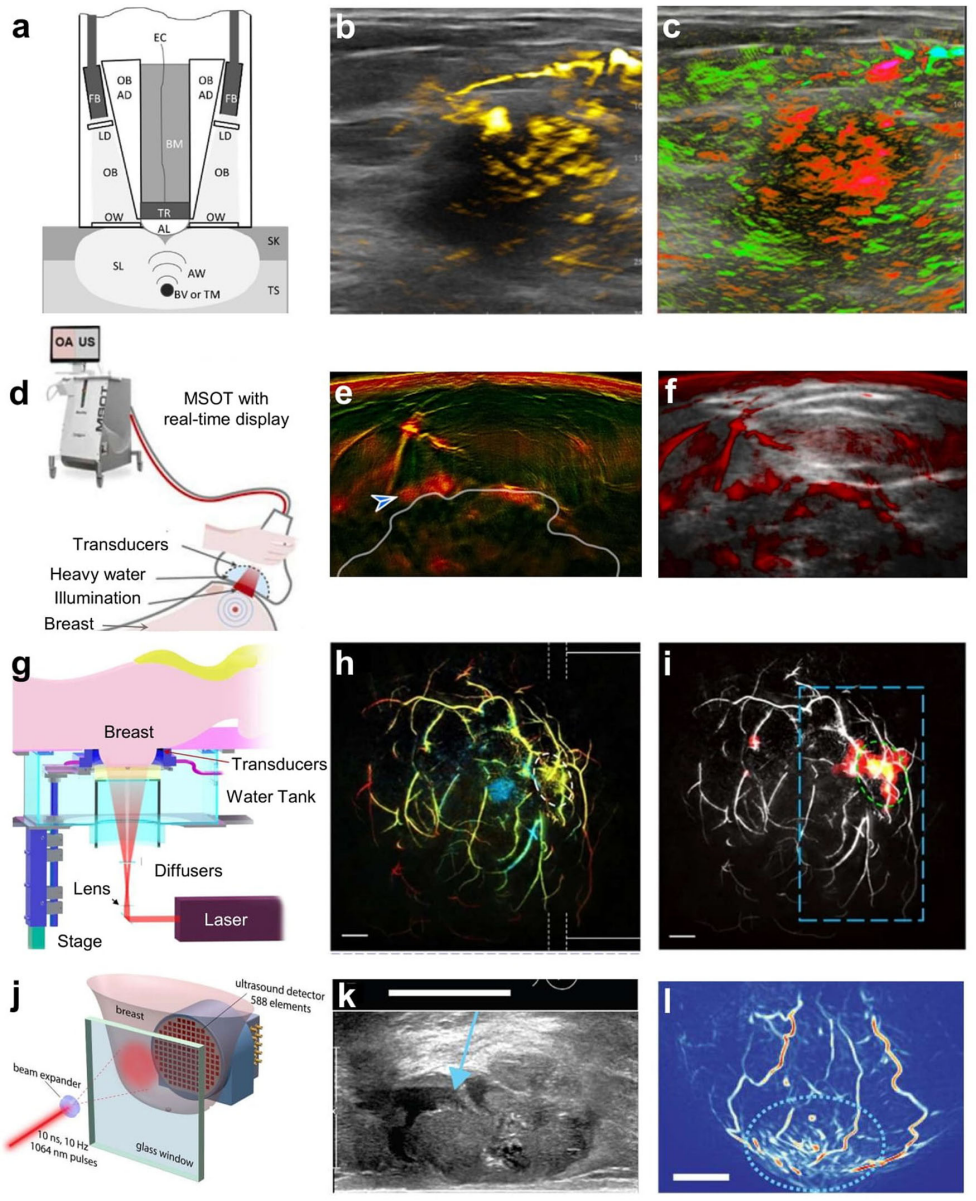
Optoacoustic (OptA) systems and representative in vivo images of breast cancer in humans. **a**–**c** Imagio® system (Seno Medical, San Antonio, USA). **a** The hand-held OptA ultrasound (US) probe for co-registered dual-modality imaging. TS tissue, SK skin, SL scattered light, OB optical beams, FB fiber bundles, LD light diffusers, OW optical windows, AW acoustic waves, BV blood vessels, TM tumors, SL scattered light, AL acoustic lens, TR transducers, EC electrical cables, BM backing material. A US image of a malignant mass is shown in gray scale with (**b**) increased internal total hemoglobin (yellow) due to high density of angiogenesis with (**c**) diffuse internal blood deoxygenation (red) within the mass and radiating parasitized feeding arteries (green). Adapted from Oraevsky et al. under a CC-BY 4.0 license^11^. **d**–**f** Multispectral Optoacoustic Tomography (MSOT) Acuity Echo (iThera Medical, Munich, Germany) system. **d** Scanner and the handheld probe. Laser light is delivered through an optical fiber bundle and a diffuser. US and OptA images are displayed on the scanner screen in real-time. Vascularization and perfusion were imaged with **e** dual-band and **f** OptA plus ultrasound (OPUS) visualization of the median of the images in the 700‍–‍740 nm range. Patches of markedly increased signal in the upper rim of a tumor are indicated by the arrow. Adapted from Kukačka et al. under a CC-BY 4.0 license^12^. **g**–**i** Single-breath-hold photoacoustic computed tomography (SBH-PACT) system. **g** Perspective cut-away view of the system. **h** Depth-encoded angiogram of an invasive ductal carcinoma (grade 2/3). **i** Automatic tumor detection on vessel density maps. The tumor is identified by a green dotted circle. Adapted from Lin et al. under a CC-BY 4.0 license^8^. **j**–**l** Twente Photoacoustic Mammoscope 2. **j** Top view schematic of the imaging tank. Adapted from Heijblom et al. under a CC-BY 4.0 license^9^. **k** US image of a mucinous carcinoma. **l** OptA maximum intensity projections in the transverse plane. Scale bars—20 mm. Adapted from Schoustra et al. under a CC-BY 4.0 license^10^.

The timing for the increasing number of OptA studies in humans in the second half of the 2010’s may be explained by key technological advances in the early 2010’s that enabled new performance specifications for OptA imaging and moved it outside the laboratory into systems that could be employed in the clinics^4^. Notable advances include the development of fast-tuning optical parametric oscillation (OPO) lasers offering more than 50 mJ of energy per pulse, enabling cross-sectional tissue images using a single laser pulse^4,5^. Tuning speeds of up to 100 Hz were reported, allowing 100 images at 100 different wavelengths per second^5^. Such lasers came with smaller form factors than laboratory systems and were tightly enclosed to prolong service times, making them suitable for use in clinical suites. This technology enabled the introduction of handheld systems that could operate without the need for averaging and, therefore, avoiding motion issues. Such illumination penetrated a few centimeters in tissue in the near-infrared (NIR: ∼650–1000 nm) and was typically coupled to ultrasound elements with central frequencies around 5 MHz, the latter typically employed in clinical ultrasonography. A further particular development was the operation of curved ultrasound arrays, first reported in 2000^6^, but only resurfaced and utilized in the early 2010’s^7^ with up to 512 ultrasound elements and corresponding analog-to-digital converters (ADCs) of the same number of parallel digitization channels. Curved array technology resulted in superior OptA imaging quality compared to using linear ultrasound arrays since the curved technology allows for the collection of more complete information from the target of interest. Finally, advanced computation models were also introduced in the early 2010’s, which offered more accurate performance than back-projection algorithms that were prevalent in the preceding decades^4^. These algorithms improve image fidelity and quantification since they are better equipped to deal with the broadband nature of the OptA signals generated in tissue following illumination with nano-second light pulses, in particular the lower frequency components, i.e., the bass of OptA music. By including lower frequencies in the reconstruction, OptA imaging moved beyond vasculature imaging to enable visualization of background structures, interfaces, and organs. Mathematically, this ability is explained by considering that larger objects, such as organ interfaces, emit lower frequencies, whereas fine microvasculature emits higher ultrasound frequencies. This technological progress eventually gave rise to commercial handheld OptA systems by the mid-2010’s, typically performing hybrid OptA and ultrasound imaging. Consequently, studies with larger number of participants virtually started in 2014–2015 and have grown constantly since then. While bed scanners have also been developed, in particular for breast cancer imaging (e.g., see Fig. 3g, j)^8–10^, the majority of OptA human imaging is based on handheld systems^11,12^.

**Fig. 4:**
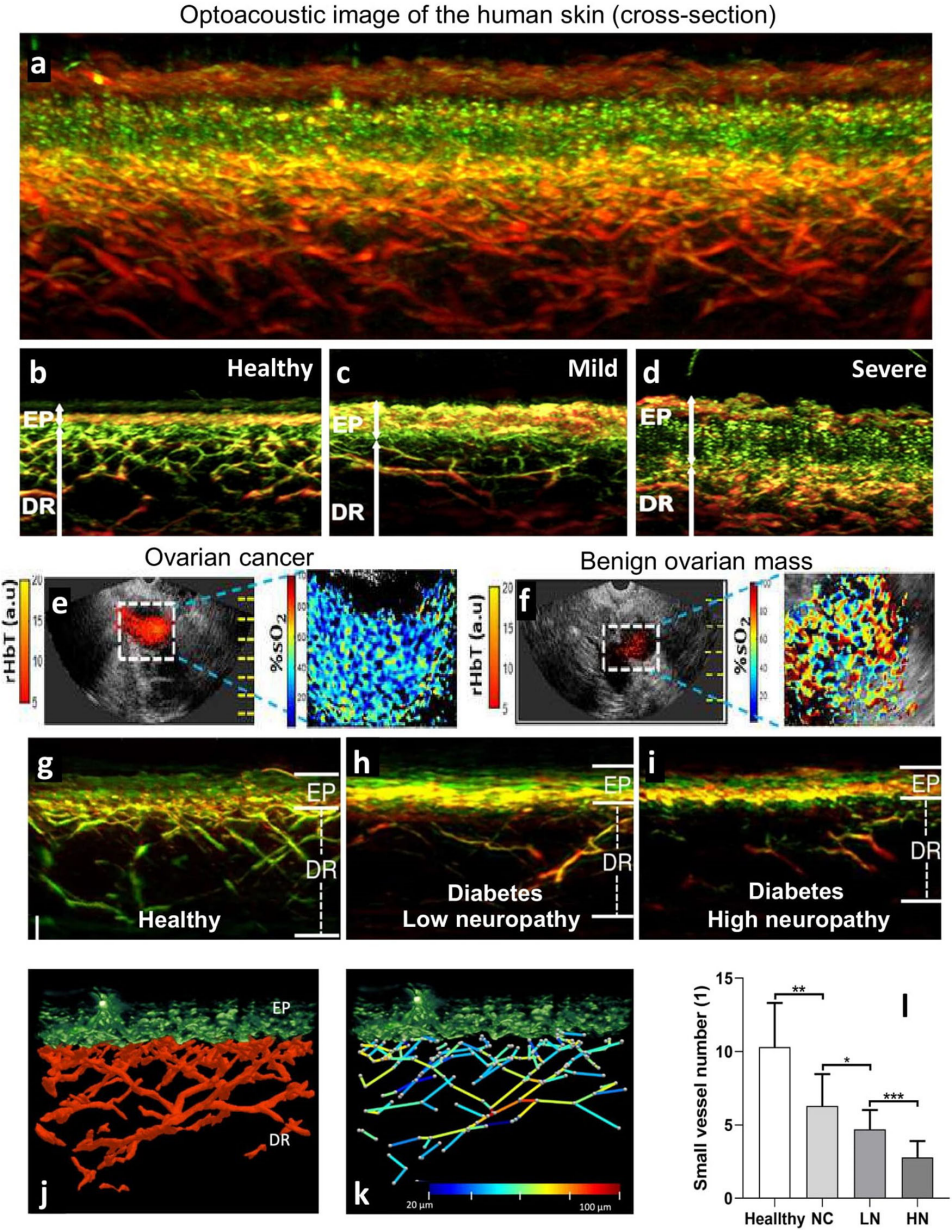
Representative in vivo mesoscopic images of healthy volunteers or patients with different diseases. **a** Cross-sectional Raster-Scan Optoacoustic Mesoscopy (RSOM) image of the human skin from a healthy volunteer, demonstrating the detail resolved at 532 nm. Green or red colors represent the high or low spatial frequencies, i.e., the finer or coarser vasculature, respectively. Fine capillary loops are seen at the epidermis as “dot”-like structures with resolutions that may reach 7 micrometers. Adapted from Aguirre et al.^20^ with permission. **b**–**d** Representative cross-sectional RSOM images of varying eczema severities. EP is the epidermal layer, whereas DR is the dermal layer. **b** Healthy volunteer, **c** patient with mild eczema, and **d** patient with severe eczema. Reprinted from Yew et al.^59^ with permission from Elsevier. **e–f** Co-registered ultrasound (US) and oxygenation saturation (%sO_2_) in ovaries with a **e** malignant or a **f** benign mass. Adapted from Amidi et al.^73^ with permission from John Wiley and Sons. **g**–**i** Example of OptA skin mesoscopy detecting systemic disease with RSOM skin images from a healthy volunteer and two diabetic patients with low and high score neuropathy. Adapted from He et al. under a CC-BY 4.0 license^49^. Segmentation of such images (**j**) could lead to an extraction of multiple features (**k**) that could classify diabetes progression. EP epidermis, DR dermis. Adapted from He et al. under a CC-BY 4.0 license^61^. **l** NC: Diabetes patients with no complications, LN: diabetes patients with low-grade neuropathy, HN: diabetes patients with high-grade neuropathy. Adapted from He et al. under a CC-BY 4.0 license^49^.

A critical development that happened at the same time frame was the development of OptA mesoscopy, i.e., systems that operate at higher frequencies using detectors with central frequencies and bandwidths in the range of tens of MHz, up to more than 100 MHz^13^. This technology only allows penetration of a few millimeters, since ultrasound attenuation by tissue becomes significant at such frequencies, however, only at resolutions at the few tens of micrometers or better. The technique allows superior images of the human skin (e.g., see Fig. 4a) and has since been employed in several studies, including studies not only of dermatologic conditions but also of endocrinology or cardiovascular medicine, revealing manifestations of systemic disease on the skin (see section “Optoacoustic macroscopy and mesoscopy”). Endoscopic implementations appropriate for human applications have also been considered using an OptA mesoscopy enclosure into a tethered capsule system, which is appropriate for scanning the gastrointestinal tract^14,15^.

We note that optoacoustic microscopy has also been described since the 1970’s^2,16^ and has seen significant growth in the past decades^17,18^. We have recently reviewed the development and operation modes of optoacoustic microscopy^19^, where we noted that while OptA macroscopy and mesoscopy yield a clear advantage over optical methods since they image deeper than optical methods, OptA microscopy does not retain this fundamental advantage. OptA microscopy operates within the same resolution and depth boundaries as optical microscopy, and therefore competes with many adept optical methods for applications. Nevertheless, references to OptA mesoscopy in the following text generally also include the clinical potential for OptA microscopy, especially when considering that at high-ultrasonic frequencies, i.e., frequencies close to 100 MHz and above, OptA methods essentially operate within the confines of OptA microscopy (i.e., depths of 1–2 mm and sub-10 micrometer resolutions).

**Fig. 5:**
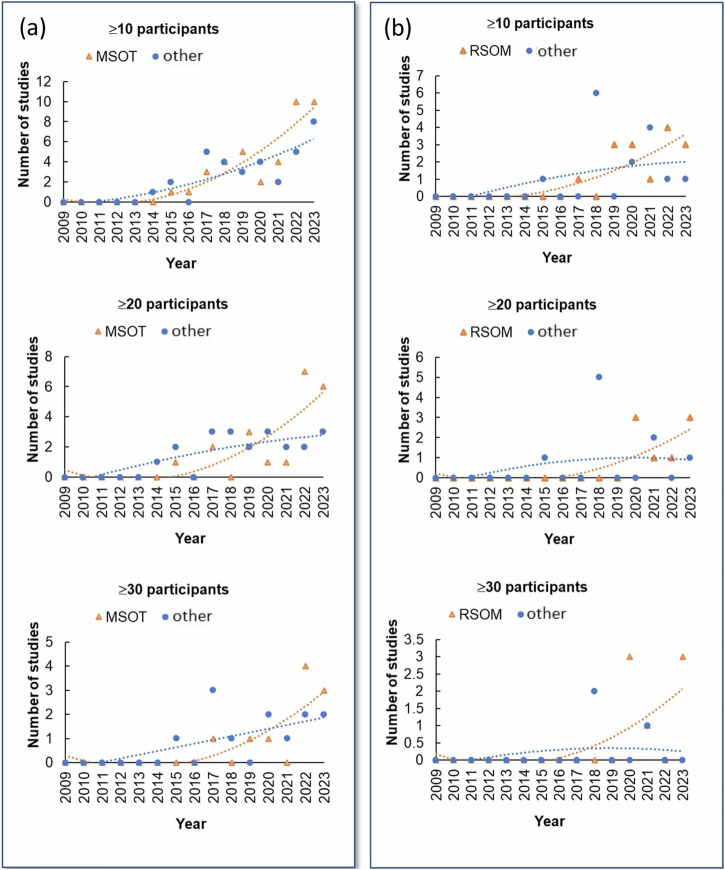
Optoacoustic studies at <10 MHz or ≥10 MHz detection frequencies. **a** The number of in vivo optoacoustic macroscopy studies at <10 MHz detection frequencies where humans were imaged using the Multispectral Optoacoustic Tomography (MSOT) platform versus all other optoacoustic imaging modalities. **b** The number of in vivo optoacoustic mesoscopy studies at ≥10 MHz detection frequencies where humans were imaged using the platform Raster-Scan Optoacoustic Mesoscopy (RSOM) versus all other optoacoustic imaging modalities.

A comparative analysis between OptA macroscopy and mesoscopy revealed that macroscopy has been employed in a much larger number of human studies so far. While 74 studies with more than 10 participants were performed at <10 MHz frequencies, only 32 studies with more than 10 participants were performed with OptA mesoscopy, i.e., with systems operating at the tens of MHz or higher. An analysis of the technology employed reveals that ~54% of macroscopic OptA studies with >10 participants have been performed on the platform Multispectral Optoacoustic Tomography (MSOT)^4^, i.e., 40 studies with more than 10 participants were performed with MSOT vs. 34 studies with all other OptA systems developed (Fig. 5a). Likewise, OptA mesoscopy has been primarily performed on Raster Scan Optoacoustic Mesoscopy^20^. Sixteen studies with more than ten participants were performed with RSOM vs. 16 studies with all other systems (Fig. 5b).

## Unique contrast mechanisms

Clinical interest in OptA imaging relates to the unique contrast enabled by the modality, in particular, optical contrast resolved in high resolution at tissue depths that reach far beyond those of optical microscopy. Optoacoustic contrast has been identified as six-dimensional^4^, since OptA imaging records three-dimensional images over time and over a spectrum (Table 2 and Supplementary Fig. 3). The 6th dimension relates to the frequency content of the OptA (ultrasound) signal when excited and recorded, which defines the scale of objects visualized and also separates macroscopy from mesoscopy.

**Table 2:** Unique label-free optoacoustic (OptA) imaging features

	Single wavelength	Spectral domain	Time domain
Raw features resolved	Hemoglobin (Hb) Vasculature Microvasculature Melanin^a^ Lipid^a^ Water^a^ Total Hb volume^b^ (THbV)	Oxygenated hemoglobin (HbO_2_) Deoxygenated hemoglobin (HbD) Melanin Lipid Water	Hemodynamic responses to stimulus (e.g., cuff occlusion, hyperthermia, cold exposure) Lipid responses to stimulus (e.g., food intake) Compartmental variance, i.e., dynamic differences in tissue compartments.
Examples of features resolved after computation	Vascular morphology features. i.e., vascular density, vasodilation, vasoconstriction, rarefaction, and many others. Inflammation burden based on THbV and vasodilation metrics	Arteries/arterioles Veins/venules Vascular and microvasculature oxygenation/Blood oxygen saturation (SO_2_) Tissue oxygenation Total Hemoglobin Volume (THbV = Hb + HbO_2_)	Endothelial and micro-endothelial function and dysfunction Aerobic metabolism/Oxygen demand (HbO_2_ to HbD rate), SO_2_ rate. Lipid circulation and lipid metabolism (rate of lipid concentration change) Tissue perfusion (THbV rate)
Clinical conditions examined	**Cancer** - Angiogenesis, abnormal vascular morphology/THbV - Melanin contrast for melanoma detection **Inflammation** - Resolve vasodilation and THbV as inflammatory patterns **Diabetes** - Assess microvascular loss and altered microvascular morphology **Cardiovascular disease** - Intraplaque hemorrhage via THbV - Lipid content - Altered vascular morphology **Dermatology** - Use inflammatory markers to quantify progression or remission in psoriasis, eczema, etc. - Quantify allergy patch test **Other** - Detect angiogenesis as indicator of healing in wounds	**Cancer** - Hypoxia/oxygen saturation for detection, characterize risk/aggressiveness **Cardiovascular disease** - Detect carotid atherosclerosis via fat-blood-ratio - Oxygenation/hypoxia in peripheral arterial disease **Arthritis** - Hypoxia or synovial hypertrophy via increased lipid content **Muscle** - Characterization of muscular dystrophy/degeneration by resolving edema, connective and fatty tissue - Exercise physiology studies via SO_2_ **Other** - Preoperative evaluation of flaps via SO_2_ - Evaluation of vascular occlusions via SO_2_ - Identify functional deficits in systemic sclerosis via artery SO_2_ - Assessing lipid content/subcutaneous fat - Characterize tissue oxygenation in various respiratory/infectious or trauma conditions	**Metabolism** - Brown adipose tissue activation - Oxygen demand - Postprandial lipid content in blood vessels **Cardiovascular disease** - Endothelial and microendothelial dysfunction as biomarkers of CVD progression **Other** - Exercise physiology based on oxygen demand - Chronic kidney disease as impairment of perfusion

^a^With appropriate single wavelength selection, it is possible to outline key molecules, however, spectral methods resolve these molecules with higher accuracy.

^b^Raw signal at an isosbestic point of Hb, HbO_2_, e.g., 805 nm.

Referring to label-free optoacoustics, single wavelength imaging in the visible and near-infrared resolves strong intrinsic tissue absorbers such as hemoglobin (Hb) and melanin. The contrast from lipids and water can also be resolved at the >900 nm spectral range. Since melanin is typically restricted to the epidermis, the contrast in the <900 nm spectral range from deeper in tissue is primarily due to Hb. With Hb concentrated in blood vessels, the most prevalent feature visualized by the OptA method is vasculature. It has been shown that due to the strong contrast and broadband nature of the OptA signal recorded, OptA imaging resolves smaller vessels and offers higher definition compared to label-free ultrasonography^21^. Besides vasculature itself, single wavelength imaging at isosbestic points, i.e., wavelengths whereby oxygenated and deoxygenated Hb absorb equally, can reveal contrast that relates to total blood (Hb) volume in each of the volume elements visualized.

Imaging at multiple wavelengths is considered for targeting or unmixing spectral contributions from different molecules. Unmixing can resolve oxygenated and deoxygenated hemoglobin, and compute maps of blood saturation in tissue. It can also unmix contributions of melanin, water, or lipids and improve their separation over single wavelength measurements by minimizing spectral cross-talk. Optoacoustics is the only modality that can visualize the distribution of oxy- and deoxy-Hb, and resolve blood oxygen saturation in tissue at high-resolution. Nevertheless, it is challenged by spectral coloring, i.e., the differential attenuation of the different wavelengths employed as a function of depth. Research is still directed toward addressing this issue. In principle, spectral methods can visualize many other light-absorbing molecules, such as collagen, myoglobin, or bilirubin. However, computing the concentrations of such additional biomolecules from the OptA data collected may result in resolving their combined contribution with hemoglobin or water, depending on the spectral similarity of the molecules resolved and the aforementioned spectral-unmixing challenges. Therefore, these molecules are not explicitly referenced in Table 2. Likewise, OptA methods can resolve contrast agents and nanoparticles, however, it is a particular advantage of optoacoustics that diverse contrast can be resolved without the use of contrast-enhancing agents, as summarized in Table 2.

The technological progress described in the “Optoacoustic studies in humans” section has now enabled fast OptA systems that can operate at video-rate imaging. Implementations of real-time imaging using single pulse illumination for each image generated have been described for more than a decade^5^, where each pulse may be at single or different wavelengths due to fast-switching wavelength tuning approaches. Therefore, OptA imaging can also capture fast dynamic phenomena, such as hemodynamic or lipid temporal responses. Finally, the particular bandwidth collected by the OptA signal defines the range of objects that can be resolved and drives macroscopy or mesoscopy implementations, as described in the “Optoacoustic studies in humans” section.

The strong contrast and image fidelity generated by vascular structures have been employed to observe vascular density in detail, extract features of vascular morphology, and act as a metric of angiogenesis and inflammation, the latter achieved by capturing increases in total blood volume or computing vasodilation. Macro- and microcirculation play key roles in maintaining tissue homeostasis, nutrient supply, and delivery of oxygen but are also implicated in disease development and linked to inflammation and degenerative processes^22^. Disease manifestation evident in the circulatory system includes impaired vascular development^23^, pathologic angiogenesis in cancer^24^, vascular adaptations related to organo-specific inflammation, immune cell recruitment^25^ and associated altered permeability^26^, and oxygen uptake^27^ or metabolism^28,29^. Hemoglobin contrast has also been employed to localize vasculature and guide non-invasive optoacoustic measurements of lipids in circulation^30^. Analyses of microvasculature changes after perturbation, such as a cuff occlusion have also been associated with endothelial dysfunction measurements^31–33^. The use of two or more fast-switching wavelengths can also resolve oxy- and deoxygenated Hb dynamics and compute the local rate of exchange between oxy- and deoxy-Hb. This rate or derivative of the local blood oxygen saturation relates to oxygen demand in tissues and has been employed for non-invasive label-free imaging of aerobic metabolism. Since no other radiological method resolves oxygenated and deoxygenated Hb or aerobic metabolism parameters, these contrast mechanisms are a particularly attractive feature of the OptA method.

## Clinical relevance of OptA features

The relation of the OptA features in Table 2 to clinical indications, as manifested in significant in-human studies, are summarized in the following text. Particular focus is placed on presenting all studies that scanned ≥30 participants, a number selected to generally indicate studies that go beyond a pilot demonstration, e.g., beyond a Phase I clinical study. Nevertheless, since the number 30 is heuristic, we also describe selected studies with a smaller number of participants (<30) that demonstrate key promising applications. In the latter case, the studies are indicated as *pilot* or *preliminary* studies.

**Fig. 6:**
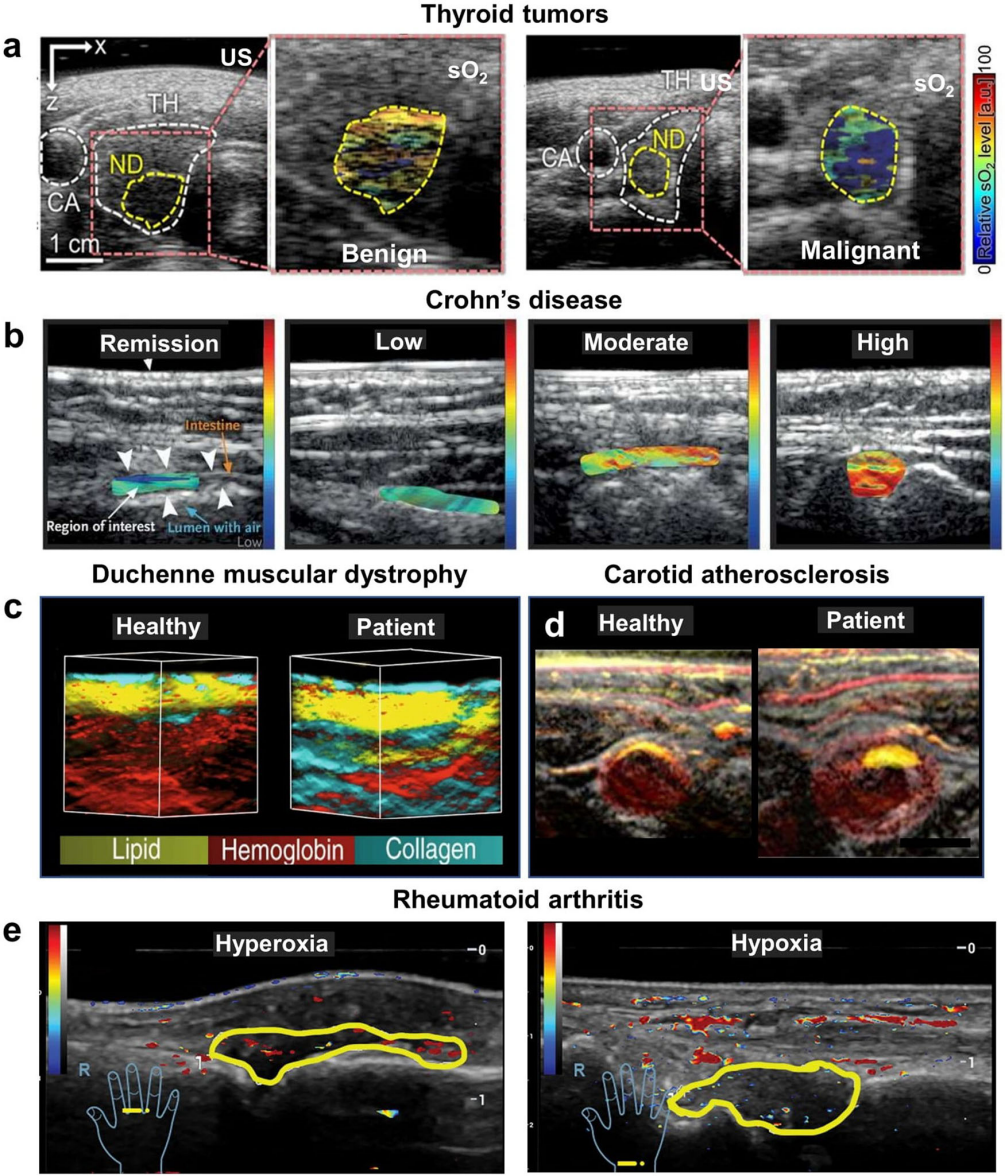
Representative in vivo macroscopy images of healthy volunteers or patients with different diseases. **a** Ultrasound (US) and co-registered optoacoustic (OptA)/US images showing hemoglobin oxygen saturation (sO_2_) in patients with a benign nodule and papillary thyroid cancer (PTC). CA carotid artery, ND nodule, TH thyroid. Adapted from Kim et al.^43^ with permission from AACR. **b** Co-registered US and OptA images showing total hemoglobin in the intestines of Crohn’s disease patients with different severity or remission. Adapted from Knieling et al.^47^. Copyright © (2017) Massachusetts Medical Society. Reprinted with permission from Massachusetts Medical Society. **c** 3D multispectral optoacoustic tomography (MSOT) images of a healthy volunteer and a patient with Duchenne muscular dystrophy. Adapted with permission from Regensburger et al.^87^. **d** Merged US/MSOT images of the carotid artery region reconstructed using prior-integrated reconstruction from a healthy volunteer and a patient with carotid atherosclerosis. Red: 850 nm, Yellow: 930 nm. Scale bar: 1 cm. Adapted with permission from Yang et al.^54^. **e** Oxygen saturation images of patients with rheumatoid arthritis reporting hyperoxia (red) and hypoxia (blue). Used with permission of Radiological Society of North America from Yang et al.^79^; permission conveyed through Copyright Clearance Center, Inc.

The majority of OptA imaging studies have been performed with visible and near-infrared wavelengths, typically resolving Hb and vascular-based contrast. Formation of images typically has employed back-projection algorithms that are simple in nature and emphasize high-frequency components, thus revealing primarily blood vessels (e.g., as seen on Fig. 3h, l). Images are often treated with vesselness filters, which beautify the appearance of vessels. It has been nevertheless shown that the application of vesselness filters while resulting in an esthetic rendering, may not always result in images that represent the imaging target with high fidelity^34^. Another representation shows OptA contrast as scattered plots of colored dots (e.g., Fig. 6a, e); owing to the difficulty of processing low-frequency components with back-projection methods, which possibly complicates interpretation. Improved fidelity and accuracy can be achieved with the use of model-based algorithms for image reconstruction^4^ and incorporation of the impulse response (point spread function) of the system employed^35^, which results in images that can also show general morphological contrast of larger interfaces and organs, not only vasculature (e.g., Fig. 6b, d).

### Hemoglobin contrast

#### Cancer

The leading demonstration using vasculature as the source of OptA contrast relates to breast cancer imaging. Given its soft tissue composition and relatively low light attenuation, the breast has been a priority target for optical and OptA imaging^36^. Consequently, the largest clinical demonstrations of OptA imaging were on breast cancer detection^3^, using handheld scanners (Fig. 3a–f). The largest OptA in-human study today, the multicenter Seno PIONEER study (*N* = 1972 patients), utilized the Imagio®, a hybrid ultrasound-OptA handheld system (Seno Medical Instruments Inc., Texas, USA)^37^. The study showed it was possible to correctly classify over 40% of suspected lesions into BIRADS (Breast Imaging Reporting and Data System^38^) 3, 4, or 5 categories, demonstrating a potential to reduce unnecessary biopsies in the future. It was also shown that certain OptA features, such as vessels extending into the suspicious lesion, the so-called “internal blush”, were associated with a higher risk of luminal versus triple-negative and HER2+ cancer subtypes^37^. Handheld hybrid ultrasound and MSOT from iThera Medical GmbH (Munich, Germany) was also employed at a single wavelength (800 nm) on 94 female participants with benign, indeterminate, or suspicious lesions^39^. A radiomic feature set was developed on 38 participants and applied to the remaining 56 patients, showing a detection sensitivity of 96.8% and a specificity of 84.6% in differentiating benign from malignant breast lesions. This performance was only possible after excluding data representative of cysts, which are known to give strong OptA contrast that may make them indistinguishable from malignant lesions^40^. The identification, and, therefore, exclusion of cysts is possible to achieve by concurrent ultrasound examination. While the studies with the highest number of participants have been performed with handheld systems, 14 studies with 2 to 42 participants have been performed in bed scanners that use mechanisms to immerse the breast in a coupling medium and surround it with detectors for whole-organ tomography^8,10,41^. Due to the larger field of view, these images typically show elaborate networks of surface vasculature (Fig. 3g–l), but the ability to visualize deep-seated contrast with high statistical power or visualize non-vascular structures has not yet been conclusively demonstrated and therefore, images are typically displayed as projections of 3D volumes (maximum intensity projections - MIPs) rather than axial or coronal individual slices as typical in handheld OptA systems (Fig. 3b, c, e, f) and other radiological modalities.

Vasculature contrast has been considered in relation to other cancers as well. Thyroid nodules have been identified in a study with 12 patients based on vascular patterns (Fig. 6a), but diagnostic performance has not yet been validated^42,43^. In pigmented skin lesions, it was found that abundant blood vessels inside the tumor area and increased vein diameter correlated with malignancy^44^. Mesoscopic OptA imaging has also been employed in cancer imaging to aid in tumor demarcation in nonmelanoma skin cancers^45^, whereas endorectal OptA imaging demonstrated imaging of recovered submucosal vasculature in rectal cancer patients responding to treatment and differentiated them from nonresponders^46^.

#### Inflammation

Inflammation results in vasodilation, which increases the hemoglobin present at sites of injury. Using total hemoglobin volume (THbV), i.e., total hemoglobin concentration as a marker of inflammation, OptA imaging using a handheld MSOT system has been used to non-invasively distinguish the grade of Crohn’s disease (Fig. 6b) in the colon of 108 patients^47^. The results were corroborated with white light endoscopy. While OptA macroscopy relies on an average vascular signal (i.e., THbV), OptA mesoscopy can resolve the vasodilation of individual cutaneous capillaries (micro-vessels). Vasodilation measurements and THbV calculation were also employed as markers of inflammation burden in psoriasis and eczema^20^ using Raster-Scan Optoacoustic Mesoscopy (RSOM). Vasodilation-based label-free signals indicative of inflammation were further employed to monitor treatment efficacy in psoriasis and demonstrated that RSOM could achieve superior detection sensitivity of treatment effect compared to the current standard of care, i.e., dermoscopy or visual assessment^48^.

#### Diabetes

Diabetes also affects microvasculature in ways different to inflammation, i.e., by reducing dermal capillary density. Due to the superior performance of high-frequency optoacoustics to resolve 10–100 micrometer diameter microvasculature^13,49,50^, RSOM images were computationally processed to resolve a large number of morphological features in epidermal and dermal vasculature and select a number of important features that could reveal the diabetes stage in studies with 115^50^ or 143^49^ participants^49^ (Fig. 4g–l). The studies point to inexpensive portable scans as the means to provide longitudinal monitoring of disease progression and showcase RSOM as a highly potent method for imaging skin microangiopathy. Such microvascular impairments may appear before the development of clinically apparent symptoms associated with systemic manifestations of the disease^51,52^.

#### Cardiovascular disease

Visualization of vasculature with contrast that is generally higher than other label-free radiological modalities has been employed to quantify aspects of cardiovascular disease. Macroscopic OptA imaging has been shown to visualize carotids^53^ and detect intraplaque hemorrhage in the carotid lumen (Fig. 6d)^54^ as an important indicator of a vulnerable plaque^55^. Combination with lipids (see next sub-section) may improve diagnostic accuracy. Hemoglobin signals have also been considered in relation to Peripheral Artery Disease (PAD) diagnostics, which manifests as inadequate blood supply in the lower extremities, ultimately leading to muscular damage^56^. It was shown that single wavelength imaging at 800 nm, largely representative of THbV, could differentiate patients with chronic limb-threatening ischemia from healthy volunteers with an AUROC of 0.88 (95% CI 0.82–0.94, *p* < 0.0001)^56^.

#### Dermatology conditions

Due to the high image quality achieved with OptA mesoscopy, in particular ultra-broadband mesoscopy such as RSOM, there are many applications directed towards clinical dermatology. In addition to visualizing the burden in inflammatory skin disease (see “Inflammation” above), RSOM quantified allergic and irritant reactions caused by patch testing through biomarkers reflecting vasodilation, vessel tortuosity, and edema^57^. Fast RSOM implementations have revealed angiogenesis patterns related to malignant skin tumors^58^. Moreover, RSOM was employed to non-invasively assess atopic dermatitis severity by enabling derivatization of an RSOM eczema severity index based on the total blood volume, vessel diameter in the dermis, and the ratio of low- and high-frequency signals in the dermis^59^ (Fig. 4b–d). Enhanced with advanced machine learning computation, differentiation between atopic dermatitis patients and healthy patients achieved 97% accuracy^60^ and accurate quantification of psoriasis severity based on automated analysis and quantification of skin features was also attained^61^.

#### Other

Systemic sclerosis has also been identified in 23 patients, but not in 19 control participants, based on vascular morphology features resolved in the nailfold^62^. However, similar features can also be generally resolved by conventional optical imaging, i.e., capillaroscopy, due to the low scattering and superficial presence of microvasculature in the nailfold. Rheumatoid arthritis (RA) has also been an OptA target using Hb contrast. Thyroid nodules have also been identified in a pilot study with 12 patients based on vascular patterns, such as vessel diameter, but usage of OptA in diagnosis has not yet been validated^42^.

### Oxygenated and deoxygenated hemoglobin

Due to the spectral nature of optoacoustics, imaging in multiple wavelengths can reveal different chromophores. In addition to melanin, oxygenated and deoxygenated Hb are strong absorbers of light in the visible spectrum and impart unique contrast to the OptA method since no other radiology method can concurrently resolve these two molecules to infer oxygen delivery, hypoxia, and associated parameters that are vital to tissue function. Although the use of multiple wavelengths adds to the complexity of the approach, revealing tissue oxygenation and possibly other spectral features is a particular strength of the OptA method.

#### Cancer

Disrupted microcirculation and hypoxia are hallmarks of cancer and may relate to cancer aggressiveness. Several macroscopic OptA breast imaging studies have employed two or more wavelengths to add functional oxygenation/hypoxia signals to the morphological contrast achieved at a single wavelength resolving Hb. Oxygenation and hypoxia relate to the notion that Hb is abundantly present in tissue and acts as an endogenous oxygen sensor, whereby oxygenated Hb indicates the presence of oxygen and de-oxygenated Hb indicates oxygen deprivation. Therefore, blood oxygen saturation (SO_2_), the ratio of oxy-Hb to the summation of oxy- and deoxy-Hb, is often reported to indicate tissue oxygenation or hypoxia, not only vascular oxygenation. Generally, SO_2_ measurements have been reported to enhance the detection of breast cancer over greyscale US^11,63–69^, whereas hypoxia was noted as a possible OptA indicator of breast cancer aggressiveness rather than a diagnostic marker^37,70^. Lower oxygen saturation was also found in ovarian epithelial cancers compared to benign lesions or normal ovarian tissue^71^, demonstrating a detection accuracy of 0.89 after the OptA measurements were enhanced with ultrasonography data. Similarly, oxygen saturation signals were shown to improve the diagnosis of malignant ovarian/adnexal lesions in 68 women referred for oophorectomy over stand-alone ultrasonography^72^. This was corroborated by a study that found that the inclusion of blood oxygenation saturation significantly improved the diagnosis of lesions and the ability to distinguish between malignant and benign masses (Fig. 4e, f)^73^. Oxygenation signals were further employed in imaging thyroid nodules for risk stratification, contributing to the detection of malignancy with 83% sensitivity and 93% specificity^43^ (Fig. 6a). Furthermore, MSOT measurements allowed differentiation between Covid-19 vaccine-associated benign lymphadenopathy and malignant lymph node metastases based on the deoxygenation level in lymph nodes^74^.

#### Cardiovascular disease

The use of dual or multiple excitation wavelengths enables functional studies in cardiovascular conditions. A well-designed monocentric diagnostic study with a derivation (101 patients) and validation cohort (96 patients) used a 3-level clinical classification (healthy, intermittent claudication, chronic limb-threatening ischemia (CLTI)) to determine the suitability of MSOT for PAD diagnostics^75^. Imaging of participants with or without peripheral artery disease before and after a walking exercise showed that oxygenated Hb content in the calf muscle was the best-performing parameter for clinical classification. Remarkably, and in contrast to MSOT, the standard measure of the ankle brachial index missed 6 (10%) of the patients diagnosed with CLTI in this study. MSOT was also shown to detect different levels of oxygenated Hb, total Hb, and oxygen saturation in patients compared to healthy volunteers^76^. Oxygenation measurements were also helpful to determine revascularization with balloon and stent angioplasty in PAD patients^76^ and identify vascular malformations^77^. Thus, in addition to risk assessment or diagnosis mentioned in the earlier section on cardiovascular disease, OptA imaging was shown able to monitor response to treatment and classify clinical stage. In a different approach, OptA mesoscopy was shown capable of resolving cutaneous microendothelial dysfunction (MiVED) at a single capillary level^61^. MiVED is recognized as an early marker of cardiovascular disease development, pointing to the use of an OptA skin measurement as the means to monitor CVD onset and progression.

#### Arthritis

Hypoxia is known to be associated with rheumatoid arthritis (RA)^78^. In a study of 118 RA patients and 15 healthy controls^79^ (Fig. 6e), and a similar study in 31 RA patients, wrist synovium hypoxia and hypoxia in the small joints correlated with less vascularization and higher disease activity and with clinical scores that reflect pain severity. Likewise, a study with 111 RA patients and 72 healthy volunteers identified a significant correlation between wrist extrasynovial tissue oxygenation and disease burden^80^. Although these findings have been collected over different anatomical locations, they indicate a relevance between OptA signals and disease-specific patterns. Such signals could lead to quantitative metrics of disease progression and possibly advance diagnostics. Likewise, approximately 30% of patients with psoriasis, a chronic inflammatory disease of the skin, also have asymmetric (oligo-)arthritis, including inflammation of an enthesis, termed psoriatic arthritis^81,82^. Dysregulated angiogenesis may play an important role even at the early phase of psoriatic arthritis, and metabolic changes in these vascular structures could lead to tissue hypoxia^83^. As such, OptA recorded changes in oxy- and deoxy-Hb levels were exploited for identifying early joint inflammation. In this regard, imaging of the finger joints in a warmed water bath found significantly higher oxygenation and THbV in 22 patients with psoriatic arthritis compared to 19 healthy controls^84^. Furthermore, a study that examined 34 patients with psoriatic arthritis, 17 patients with rheumatoid arthritis, and 36 healthy volunteers found that enthesitis-related sonographic changes were associated with increased total Hb, oxygen saturation, and collagen, whereas arthritis-related clinical and ultrasonography findings were related to increased THbV, reduced oxygenation and reduced water (and collagen) signals^85^. The study points to OptA measurements resolving distinct metabolic differences between arthritis and enthesitis in humans in vivo^85^.

#### Muscle

Using model-based approaches (see the introduction of section “Unique contrast mechanisms”), OptA imaging can offer imaging of morphology, including subcutaneous fat and the superficial parts of the muscle, not only vasculature. Applied to inherited neuromuscular diseases, in particular Duchenne muscular dystrophy (DMD), OptA morphology imaging visualized changes associated with the progressive replacement of muscle tissue by connective and fatty tissue^86^. In this case, the unmixed Hb signal, which also contains contributions from myoglobin, decreased in patients compared to healthy participants and the decrease in patients was correlated with pathophysiological muscular degeneration^87^ (Fig. 6c). Similarly, reduction of hemoglobin signals was correlated to neurogenic muscle degeneration, so-called spinal muscular atrophy (SMA)^88^, meaning that OptA could possibly be used to identify treatment effects in the future^89^.

#### Other

Tissue oxygenation is involved in many other conditions, from exercise physiology to circulatory pathologies. Optoacoustic imaging was considered for improving preoperative evaluation of tissue flaps, in particular for identifying sites of functional vasculature and differentiating between arteries and veins using 756 nm and 797 nm^90^. Elevated oxygenation and angiogenesis were employed as indicators of wound healing^91^ including in chronic leg ulcers after topical application of Hb^92^. The Raynaud’s phenomenon^93^ was also recorded using OptA imaging by resolving a drop in baseline oxygenation in fingers and capturing the hyperemic oxygenation response following occlusion and release in systemic sclerosis patients compared to healthy volunteers^94^.

### Melanin

Being a strong light absorber, melanin has been employed as OptA contrast to detect melanoma metastasis in lymph nodes^95^. However, the requirement to detect even a single metastatic cell in the lymph nodes may limit the in vivo application of the method and render it more suitable for fast scanning of excised nodes. Spectral unmixing was also used to resolve distributions of melanin and oxy-hemoglobin signals in nonmelanotic skin cancers in 26 patients using real-time volumetric MSOT imaging based on a array of detectors arranged in half-sphere geometry, showing a good correlation between non-invasive OptA findings and histological analysis on tissue specimens. However, the study reported that the use of such spherical detectors may not achieve the depth required and may compromise the accuracy of detection. Moreover, the use of melanin in amelanotic cancers may not be the optimal selection of contrast^96^. Optoacoustic mesoscopy has also been applied to image melanoma and could enable non-invasive determination of melanoma depth and the underlying angiogenesis with high precision^58^.

### Lipids and water

Lipid sensing is another potentially highly attractive feature of OptA imaging as it relates to nutritional studies and several pathologies. In a pilot study, macroscopic OptA was employed for non-invasive and longitudinal postprandial lipid measurements in arteries, veins, and muscle after study participants orally ingested a lipid-rich meal^30^. Even though, in the current implementation, it was not shown to be possible to spectrally differentiate particular lipid species, the study points to new means of performing disseminated studies for continuous tracking of lipid dynamics in real time, as it may be associated with novel diagnostics in nutritional studies and cardiometabolic conditions. Lipid-to-Hb ratios from lesions in the carotid lumen, resolved by MSOT (Fig. 6d), were also shown to differentiate between patients with carotid atherosclerosis and healthy volunteers (*p* < 0.05)^54^. Lipid signals were also considered for identifying adipose tissue, in particular brown fat, and spatially separating it from muscle tissue^97^. Lipid signatures were also explored in the study of synovial hypertrophy, a condition that was associated with increased lipid content in the joints of patients with psoriatic or rheumatoid arthritis compared to healthy volunteers^85^.

Water can also be resolved by OptA measurements, typically at wavelengths longer than 960 nm, but has not been broadly explored in the studies we identified. Nevertheless, notable references include the study of edema or higher water presence in association with muscle degeneration and fibrosis^87^ and in relation to changes observed in supraclavicular brown fat activation after participants were exposed to cold^97^.

### Rate of hemoglobin and SO_2_ change as indicators of perfusion and metabolism

Optoacoustic methods can resolve Hb, vasculature, oxygenation and SO_2_ contrast with video frame rates, owing to the technological progress discussed in the “Optoacoustic studies in humans” section. Therefore, the method is well suited to sense hemodynamics, the vasometabolic coupling that relates metabolism to flow rate, oxygen demand, and other dynamic phenomena. Fast systems also demonstrated concurrent imaging at multiple wavelengths in the tens of Hz frame rates^5^. Such spatiotemporal-spectral imaging, uniquely achieved by fast optoacoustics, was shown to detect changes in SO_2_ rate and resolve activation of brown adipose tissue (BAT) in humans following exposure to cold^97^.

Fast video-rate imaging can also be employed to compute perfusion, i.e., the rate of THbV monitored at an isosbestic point of hemoglobin. Alternatively, THbV can be computed as the summation of oxygenated and deoxygenated Hb. Blood flow and tissue perfusion can also be resolved by ultrasonography and other radiological methods, however, optoacoustics can independently visualize perfusion of oxy- vs. deoxy- Hb when using multi-wavelength optoacoustics and also resolve the end-result of perfusion in tissue, i.e., tissue oxygenation. The combination of tissue oxygenation and perfusion measurements using OptA macroscopy has been used to visualize muscle perfusion and oxygenation under exercise^98^ or differentially resolve patterns of oxy- and deoxy-Hb in response to arterial or venous occlusions^99^. Aspects of cardiovascular disease were also resolved dynamically with flow-mediated dilatation studies during the course of an arterial occlusion test, which enables the characterization of endothelial function^100^. Optoacoustic mesoscopy has also demonstrated the ability to resolve various temporal biomarkers associated with microendothelial dysfunction of cutaneous microvasculature in PAD patients following a cuff occlusion^76^.

### Exogenous agents

The overwhelming majority of OptA in-human studies have been based on endogenous contrast. Nevertheless, a number of studies have employed exogenous agents for contrast enhancement. Lymph nodes and lymphatic vessel depth have been resolved by OptA macroscopy using the absorption contrast of the FDA-approved fluorochrome Indocyanine Green^101^. In a pilot study, intestinal transit times were also determined by an oral application of ICG^102^. As the dye was excreted almost unchanged, it could be used as a radiation-free alternative to fluoroscopic examinations. Other studies have employed targeted fluorescent agents, such as fluorescently labeled therapeutic antibodies bevacizumab for visualizing carotid atherosclerosis^103^, or panitumumab^104^ and cetuximab^105^ for cancer imaging. Fluorescence labels are however optimized for fluorescence emission, not OptA signal generation. Moreover, studies utilizing targeted agents are faced with visualizing labels at much lower concentrations than studies using ICG. Consequently, these early studies did not deliver conclusive findings. The development of contrast agents with high absorption cross-sections optimized for OptA imaging may lead to better contrast enhancement but the particular benefits over other radiological modalities also using contrast agents must be established.

## Challenges and opportunities

### Macroscopy

The major OptA challenge remains the penetration depth of the OptA method. The depth depends on the optical properties of the tissue at the wavelength employed and the ultrasound frequency used. However, in most cases, the maximum penetration depth does not exceed ~3 cm of penetration in many tissues. The relative sensitivity of OptA imaging as a function of depth, especially for claims of tissue penetration of more than 3 cm, should be rigorously validated on a per-implementation basis. Therefore, OptA imaging does not currently match the penetration limit of ultrasonography. This limitation restricts the use of optoacoustics to applications of relatively superficial disease manifestations. Another limitation is that macroscopic OptA imaging generally requires expensive light sources that may reach 6-figure prices in euros or dollars. Therefore, the systems are offered at much higher price brackets than ultrasound imaging devices. Optoacoustic macroscopy is still offered at only a fraction of the cost of other common radiological modalities such as Magnetic Resonance Imaging (MRI) and Positron Emission Tomography (PET), but at a 6-figure cost, dissemination in point-of-care facilities will impose an economic burden. It is, therefore, possible that only larger medical facilities can implement the technique in routine clinical examinations, similar to other radiological modalities. In terms of safety, the use of light energies within regulated limits does not impose a high risk for general human health, however, clinical application of the technique requires the consideration of protection from laser light in terms of eye safety. For this reason, commercial OptA systems come with enclosures which do not allow open beams and laser switches that allow lasing only when the scanner is in contact with tissue. Depending on the implementation, the use of specially designed laser protection measures, such as eye-protection goggles, may be necessary while using laser-driven systems.

A third critical limitation of the technology relates to the spectral performance, in particular to spectral coloring, which, as briefly mentioned in the “Optoacoustic macroscopy and mesoscopy” section, is a term that implies the different rates of attenuation of light at different wavelengths as it propagates through tissue. Spectral coloring alters the apparent spectra of chromophores as a function of depth and the spatial variation of tissue optical properties. Since the optical properties of tissue are not known and are notoriously challenging to compute, an exact solution to the problem of spectral coloring remains an unsolved computational problem. Nevertheless, research into three-dimensional non-linear spectral unmixing has yielded potent advances in the past years, in particular when the spectral unmixing problem is transformed from the spatial to the spectral domain^106,107^. Accurate computational methods for OptA spectral unmixing remain a topic of research, and we expect that advances with newer computational tools will converge to accurate solutions in the near future. Skin color may also affect the OptA measurement, however, since this attenuation term is not distributed in volume but is contained at the very superficial melanin layer on the skin, it only acts as an attenuation factor. This means that high melanin concentration attenuates the signal and reduces the depth reached but does not contribute significantly to spectral coloring. Besides acquiring for a longer time to compensate for signal loss, beamforming methods have been proposed to mitigate the effects of varying melanin concentrations on an OptA image^108^. In relation to the clinical applications considered, a challenge for the translation of macroscopic OptA is the availability of other established radiological modalities. For example, in its most prevalent application (i.e., breast cancer imaging), X-ray mammography, MRI, and ultrasonography achieve high detection sensitivity and specificity, yielding a barrier to the introduction of a new modality. Even if OptA imaging uses safe light energies and can be portable and economical compared to X-ray or MRI, it will need to conclusively demonstrate a compelling feature in the detection of breast cancer or other cancers not served today by the standard of care. One such candidate feature is the ability to follow treatment and record Hb signals as markers of functioning vascularization, oxygen delivery, dysregulated cellular energetics, or tumor-promoting inflammation^109–112^.

Overall, the ability to resolve tissue oxygenation and oxidative, and lipid metabolism are unique features of the OptA modality and may become useful as there is a need for portable readings relating to the cardiometabolic spectrum of diseases, in particular for characterizing muscle, adipose tissue, and other tissue energetics, lipid distribution, and circulation. Optoacoustic imaging in the field of muscle imaging has shown significant advancement, and research has progressed beyond feasibility studies. PAD diagnostics have also advanced and consistent results have been presented by independent groups. Macroscopic OptA imaging may help to identify patients who are currently missed by other diagnostics. Moreover, it may be helpful in the workup of patients with unclear leg pain where ischemic or hereditary genesis needs to be ruled out, but such ability requires further clinical validation. Another possible application is diagnosing and monitoring of diabetes through its angiopathic involvement with macroscopic OptA imaging at an early stage. The oxidative capacity of tissues, particularly in connection with aspects of sports medicine, may also be an ideal field of study. In this field of research, there are overlaps with MRI, however, OptA imaging can become significantly more disseminated as it comes with markedly lower infrastructure and implementation costs than MRI. Finally, the ability to detect lipids and water without the need for contrast agents opens a window of opportunities in nutritional sciences and the development of personalized profiles associated with meal processing and associated measurements of energetics.

### Mesoscopy

Despite the promising outlook described in the previous sections, OptA macroscopy faces several challenges associated with spectral unmixing as a function of depth, the cost of macroscopic implementations, and the availability of many other radiological methods that are already established and penetrate deeper. Conversely, a unique characteristic of the OptA modality is the particular implementation in the mesoscopy regime, which offers visualization of optical contrast with resolutions in the tens of micrometers or better, much deeper than optical microscopy. Therefore, it serves a particular niche for a multitude of conditions of local and systemic disease that manifest superficially, i.e., at skin layers or under the epithelium. When imaging the skin, melanin concentration can affect the signal-to-noise in the images^113^, similar to effects seen in macroscopy. However, due to the more superficial depths imaged in mesoscopic mode, the effects of melanin may be compensated for even more efficiently than in macroscopy by varying the averaging times or adjusting the delivered energy.

Compared to OptA macroscopy, mesoscopy implementations come at a fraction of the cost and are not affected significantly by spectral coloring due to the superficial depths probed. Although a larger volume of OptA studies have been performed at the macroscopic regime (*N* = 208), the emergence of mesoscopic imaging is evident (*N* = 110) and growing at a similar rate as macroscopic studies (Fig. 5). Mesoscopy leads to unprecedented detail in imaging the skin layers and resolving features under the epithelium, yielding unique cross-sectional images unmatched by any other method available (e.g., Fig. 4a). Therefore, it can be used for detailed morphological, functional, and molecular imaging when measurements are performed over wavelength and over time. Besides the well-demonstrated applications in dermatology discussed in the preceding sections, a major advance of the technology is the recent demonstration of detecting established links between measurements in the human skin and systemic conditions such as diabetes and cardiovascular disease^49,50,114^. Likewise, developments of highly-performing optoacoustic endoscopes^14,15^ point to future porting of the technology to interventional procedures. We expect that these developments in OptA mesoscopy will lead to critical future applications of the technology, especially due to the advantages offered by its portability and low cost.

## Summary and outlook

There has been significant and constant technological progress since the first in vivo demonstrations of OptA imaging in animals^115^ in 1999, and in humans^3^ in 2001. The introduction of robust technology by the mid-2010’s and subsequent commercial availability of hand-held OptA systems for macroscopy and mesoscopy has driven OptA outside the realm of academic laboratories or pilot studies on a few participants to the hands of clinicians and researchers who prioritize technology utilization rather than technology development (see Supplementary Table 1). This shift drove marked growth in the number of in-human OptA studies in less than a decade, and exploration of various abilities, unique features and applications of the OptA modality.

Despite its overwhelming use in cancer imaging, as manifested in Fig. 2, applications relating to the cardiometabolic syndrome may be a field better suited to OptA clinical deployment. Tumor imaging based on hemoglobin and hypoxia signals has not yet shown compelling diagnostic or theranostic performance. Conversely, the ability to visualize hemodynamics, tissue oxygenation and oxygenation rates, and lipid dynamics are requisites for a spectrum of measurements ranging from nutritional studies, exercise physiology, and cardiovascular conditions, as discussed in the “Clinical relevance of OptA features” section. Importantly, the portability and non-invasive nature of the technology can enable a distributed placement of the modality for assessing such critical functional and molecular signatures at point-of-care facilities, increasing the potential to study a broader population than the one allowed by powerful but expensive and low-throughput modalities such as MRI or PET, the latter also using radiation and therefore not suited for longitudinal studies or studies of a non-patient population^116^.

OptA mesoscopy, in particular, may find compelling applications in assessing dermatological and systemic conditions in the skin. OptA mesoscopy may be the only method that can non-invasively determine primary melanoma depth and extend visualization to several millimeters under the skin surface, possibly leading to a reduction of unnecessary biopsies. In addition, quantitative measurements of inflammatory skin disease could render OptA as the method of choice for improving diagnostics and treatment efficacy in dermatology. Critically, new applications based on measuring skin biomarkers of systemic diseases may offer powerful ways to monitor early disease development, disease progression and intervention efficacy in the future. Such detailed readings are also necessary for prevention programs to detect early disease phenotypical changes, informing not only on risk but also on the actual condition of the monitored individual.

OptA mesoscopy could also become of interest in endoscopic and interventional applications, especially due to the recent development of capsule OptA endoscopy systems that allow for sub-surface label-free identification of blood vessels and other morphological changes due to the development of disease, as well as the visualization of tissue oxygenation or inflammation. This new ability could be offered as a stand-alone modality or in combination with other endoscopic methods, such as white light endoscopy or endoscopic optical coherence tomography, to offer a more comprehensive view of the targeted organ. Besides applications in endoscopic visualization of the gastro-intestinal tract, many other interventional procedures could benefit from endoscopic OptA, including applications in brain surgery or plastic surgery to assess tissue viability or assess functional characteristics in pancreatic surgery.

While the studies presented demonstrate the growing importance of OptA imaging, we are still in the early days of clinical propagation. Nevertheless, due to the compelling and unique characteristics of the method, there is a strong drive for clinical adoption. Efficient clinical translation of optoacoustics would require several next steps, including the regulatory approval of commercial systems, quality control abilities that ensure reproducibility of the findings between clinical centers, and processing algorithms that offer accuracy in the markers resolved. Another consideration to be fulfilled is that different user groups (doctors, radiologists, internists, technicians) may have different requirements and expectations on system design and performance, which will have to be addressed with care.

## Supplementary information

Supplementary Information

## Data Availability

No datasets were generated or analyzed during the current study.
