# Supplementary material for: A primer on current status and future opportunities of clinical optoacoustic imaging
Source: Npj Imaging. 2025 Jan 27;3:4. doi: 10.1038/s44303-024-00065-9 (PMC12091691; doi:10.1038/s44303-024-00065-9)
Supplement: Supplementary file 1 — Supplementary Information [file 44303_2024_65_MOESM1_ESM.pdf]

Supplementary Information

Supplementary Table 1. Optoacoustic systems used in studies with the largest patient cohorts. Abbreviations: CE - Conformité Européene, EU MDR – European Union Medical Device Regulation, FDA PMA – US Food & Drug Administration premarket approval, IRB – Institutional Review Board, MSOT – Multispectral Optoacoustic Tomography, NCT – National Clinical Trial, RSOM – Raster-Scan Optoacoustic Mesoscopy.

Disease examined	Clinical trial information	Patient number	Name of system	Approval	Reference
Breast cancer	Seno-pioneer trial	1972	Imagio	FDA PMA	1
Breast cancer	NCT01943916	1790	Imagio	FDA PMA	2
Breast cancer	NCT04030104	480	Imagio	FDA PMA	3
Peripheral artery disease	NCT04641091	197	MSOT Acuity Echo	CE/EU MDR	4
Peripheral artery disease	NCT04641091	193	MSOT Acuity Echo	CE/EU MDR	5
Rheumatoid arthritis	None. IRB approval only.	183	Custom-built system	-	6
Diabetes	None. IRB approval only.	143	Custom-built RSOM system	-	7
Rheumatoid arthritis	NCT04297475	133	Custom-built system	-	8
Breast cancer	None. IRB approval only.	120	Custom-built system	-	9
Breast cancer	None. IRB approval only.	119	Custom-built system	FDA PMA	10
Diabetes	None. IRB approval only.	115	Custom-built RSOM system	-	11
Psoriasis	None. IRB approval only.	110	Custom-built RSOM system	-	12
Breast cancer	IRB-approved phase 3 pilot study	100	Imagio	FDA PMA	13
Breast cancer	None. IRB approval only.	94	MSOT Acuity Echo	CE/EU MDR	14
Breast cancer	IRB-approved phase 3 pilot study	92	Imagio	FDA PMA	15
Atopic dermatitis	None. IRB approval only.	91	RSOM Explorer C50 System	-	16
Crohn's disease	NCT02622139	91	MSOT Acuity Echo	CE/EU MDR	17
Rheumatoid and psoriatic arthritis	None. IRB approval only.	87	MSOT Acuity Echo	CE/EU MDR	18
Melanoma	IRB approval. Registered at the German Clinical Trials Register.	83	MSOT Acuity Echo	CE/EU MDR	19
Atopic dermatitis	None. IRB approval only.	76	RSOM Explorer C50 System	-	20

Supplementary Figure 1. Graphs summarizing the growth of in-human optoacoustic studies with different numbers of participants. (a) All studies since 2009, even studies with 1 participant. (b) Studies with more than or equal to 10, 20, 30, or 40 participants plotted together. (c) Studies with 10-19, 20-29, 30-39, or ≥40 participants plotted together.

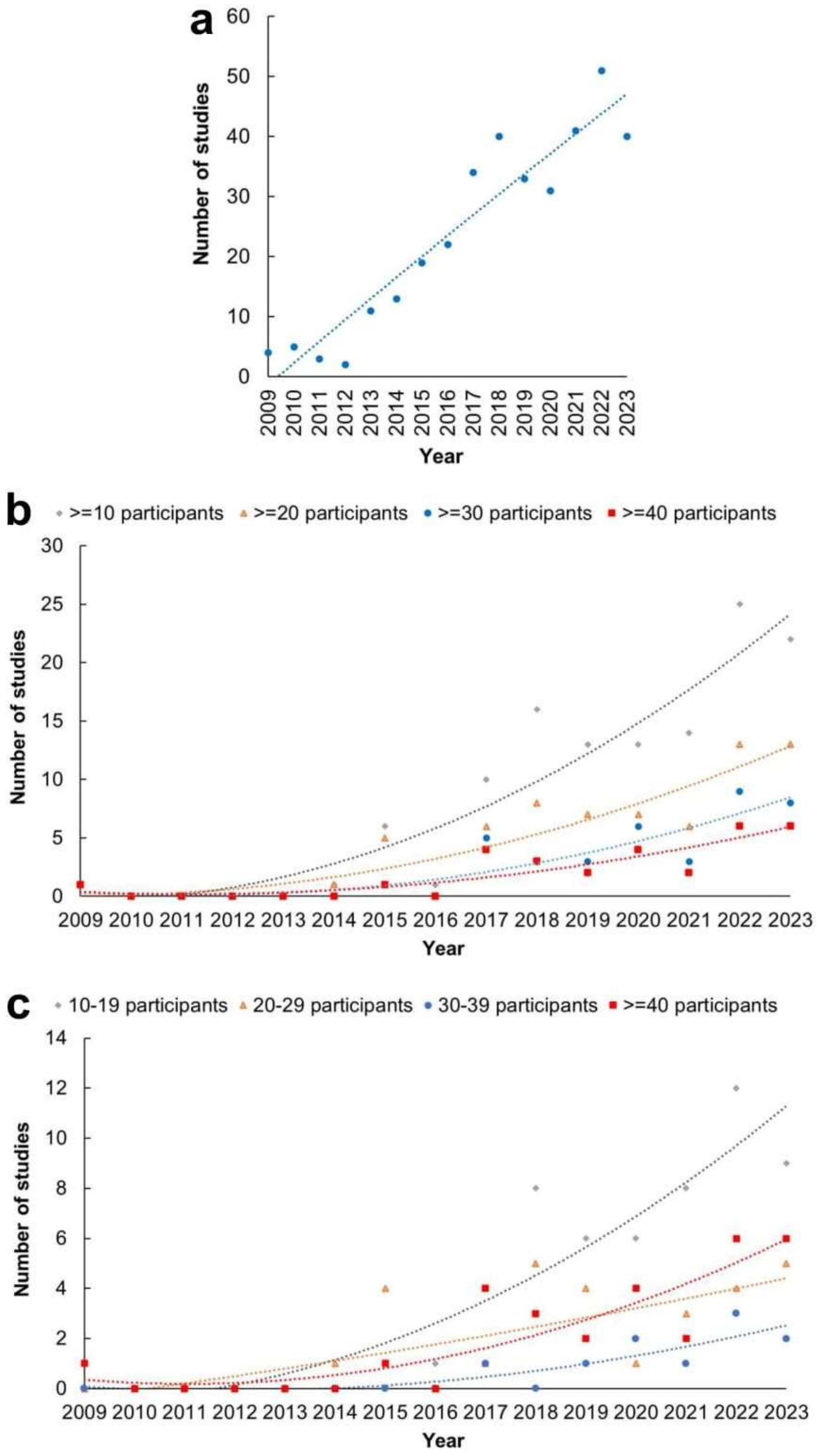

Supplementary Figure 2. Histogram showing numbers of in vivo optoacoustic studies in humans as a function of the number of participants. Each column is divided into studies with patients (blue) or only healthy volunteers (grey).

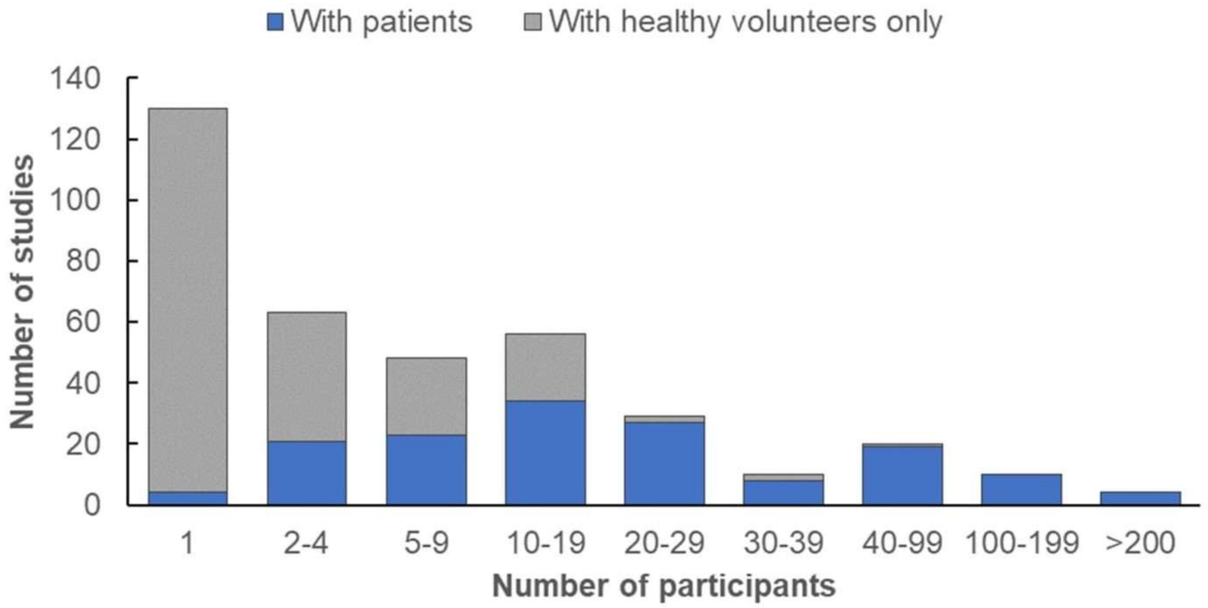

Supplementary Figure 3. Number of excitation wavelengths utilized in optoacoustic studies in humans in vivo.

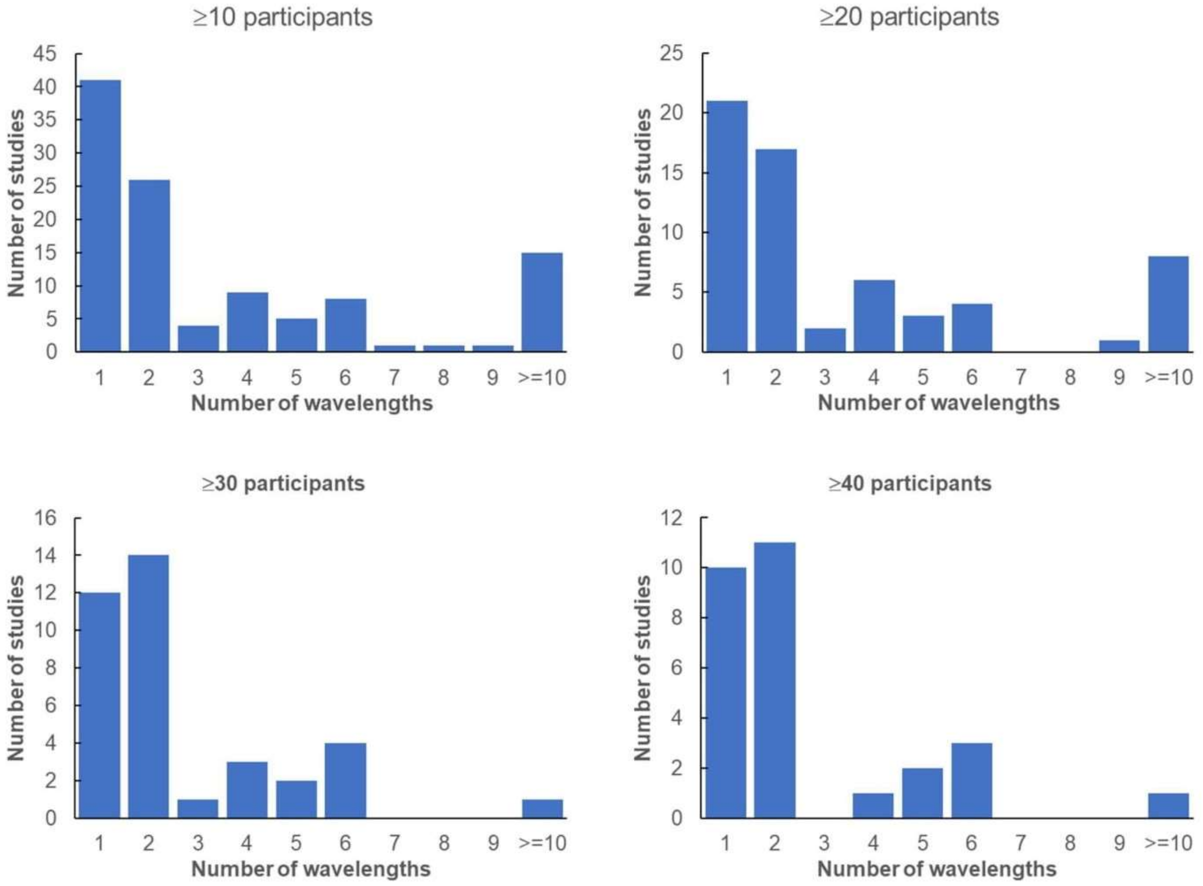

Supplementary References

1 Dogan, B. E. *et al.* Optoacoustic Imaging and Gray-Scale US Features of Breast Cancers: Correlation with Molecular Subtypes. *Radiology* **292**, 564-572 (2019). https://doi.org:10.1148/radiol.2019182071

2 Neuschler, E. I. *et al.* A Pivotal Study of Optoacoustic Imaging to Diagnose Benign and Malignant Breast Masses: A New Evaluation Tool for Radiologists. *Radiology* **287**, 398-412 (2018). https://doi.org:10.1148/radiol.2017172228

3 Seiler, S. J., Neuschler, E. I., Butler, R. S., Lavin, P. T. & Dogan, B. E. Optoacoustic Imaging With Decision Support for Differentiation of Benign and Malignant Breast Masses: A 15-Reader Retrospective Study. *American Journal of Roentgenology* **220**, 646-658 (2023). https://doi.org:10.2214/ajr.22.28470

4 Günther, J. S. *et al.* Targeting Muscular Hemoglobin Content for Classification of Peripheral Arterial Disease by Noninvasive Multispectral Optoacoustic Tomography. *JACC: Cardiovascular Imaging* **16**, 719-721 (2023). https://doi.org:https://doi.org/10.1016/j.jcmg.2022.11.010

5 Träger, A. P. *et al.* Hybrid ultrasound and single wavelength optoacoustic imaging reveals muscle degeneration in peripheral artery disease. *Photoacoustics* **35**, 100579 (2024). https://doi.org:https://doi.org/10.1016/j.pacs.2023.100579

6 Huang, Z. *et al.* Correlation between the oxygenation status of extrasynovial tissue in the wrist and disease activity in rheumatoid arthritis: a photoacoustic imaging study. *Rheumatology*, keae047 (2024). https://doi.org:10.1093/rheumatology/keae047

7 He, H. *et al.* Opening a window to skin biomarkers for diabetes stage with optoacoustic mesoscopy. *Light: Science & Applications* **12**, 231 (2023). https://doi.org:10.1038/s41377-023-01275-3

8 Yang, M. *et al.* Synovial Oxygenation at Photoacoustic Imaging to Assess Rheumatoid Arthritis Disease Activity. *Radiology* **306**, 220-228 (2023). https://doi.org:10.1148/radiol.212257

9 Huang, Z. *et al.* Assessment of Oxygen Saturation in Breast Lesions Using Photoacoustic Imaging: Correlation With Benign and Malignant Disease. *Clinical Breast Cancer* **24**, e210-e218.e211 (2024). https://doi.org:10.1016/j.clbc.2024.01.006

10 Li, G. *et al.* Photoacoustic Imaging Radiomics to Identify Breast Cancer in BI-RADS 4 or 5 Lesions. *Clinical Breast Cancer* **24**, e379-e388.e371 (2024). https://doi.org:https://doi.org/10.1016/j.clbc.2024.02.017

11 Karlas, A. *et al.* Dermal features derived from optoacoustic tomograms via machine learning correlate microangiopathy phenotypes with diabetes stage. *Nature Biomedical Engineering* **7**, 1667-1682 (2023). https://doi.org:10.1038/s41551-023-01151-w

12 He, H. *et al.* Machine Learning Analysis of Human Skin by Optoacoustic Mesoscopy for Automated Extraction of Psoriasis and Aging Biomarkers. *IEEE Transactions on Medical Imaging* **43**, 2074-2085 (2024). https://doi.org:10.1109/TMI.2024.3356180

13 Neuschler, E. I. *et al.* Downgrading and Upgrading Gray-Scale Ultrasound BI-RADS Categories of Benign and Malignant Masses With Optoacoustics: A Pilot Study. *American Journal of Roentgenology* **211**, 689-700 (2018). https://doi.org:10.2214/ajr.17.18436

14 Abeyakoon, O. *et al.* An optoacoustic imaging feature set to characterise blood vessels surrounding benign and malignant breast lesions. *Photoacoustics* **27**, 100383 (2022). https://doi.org:https://doi.org/10.1016/j.pacs.2022.100383

15 Butler, R. *et al.* Optoacoustic Breast Imaging: Imaging-Pathology Correlation of Optoacoustic Features in Benign and Malignant Breast Masses. *American Journal of Roentgenology* **211**, 1155-1170 (2018). https://doi.org:10.2214/ajr.17.18435

16 Yew, Y. W. *et al.* Raster-scanning optoacoustic mesoscopy imaging as an objective disease severity tool in atopic dermatitis patients. *Journal of the American Academy of Dermatology* **84**, 1121-1123 (2021). https://doi.org:10.1016/j.jaad.2020.06.045

17 Knieling, F. *et al.* Multispectral Optoacoustic Tomography for Assessment of Crohn's Disease Activity. *New England Journal of Medicine* **376**, 1292-1294 (2017). https://doi.org:doi:10.1056/NEJMc1612455
18 Tascilar, K. *et al.* Non-invasive metabolic profiling of inflammation in joints and entheses by multispectral optoacoustic tomography. *Rheumatology* **62**, 841-849 (2022). https://doi.org:10.1093/rheumatology/keac346
19 Stoffels, I. *et al.* Assessment of Nonradioactive Multispectral Optoacoustic Tomographic Imaging With Conventional Lymphoscintigraphic Imaging for Sentinel Lymph Node Biopsy in Melanoma. *JAMA Network Open* **2**, e199020-e199020 (2019). https://doi.org:10.1001/jamanetworkopen.2019.9020
20 Park, S. *et al.* Model learning analysis of 3D optoacoustic mesoscopy images for the classification of atopic dermatitis. *Biomed. Opt. Express* **12**, 3671-3683 (2021). https://doi.org:10.1364/BOE.415105
